# PB.32. Type of breast surgery in patients undergoing neoadjuvant chemotherapy: role of DCE-MRI

**DOI:** 10.1186/bcr3743

**Published:** 2014-11-03

**Authors:** M Halim, M Sreenivas

**Affiliations:** 1 UHCW NHS Trust, Coventry, UK

## Introduction

The aim was to assess the role of MRI in neoadjuvant chemotherapy and whether this has helped the surgical decision. A secondary aim was to identify the pattern of malignant lesions seen on MRI and to correlate this with the surgical decision.

## Methods

Twenty-seven consecutive patients who had MRI scans prior to neoadjuvant chemotherapy were identified. Nineteen patients had a postchemotherapy scan.

The pattern of malignant lesions was identified on the baseline MRI and divided into five previously described categories (circumscribed (type 1), nodular (type 2), diffuse (type 3), patchy enhancement (type 4) and septal spread (type 5)).

## Results

Out of the 27 patients, 11 patients had mastectomies and 16 underwent breast-conservation surgery (BCS). From the 19 patients who had a postchemotherapy MRI, four had complete therapeutic response, 14 had partial response and one showed disease progression. Of the 27 patients, 14 had circumscribed malignancy with an average size of 39 mm (range = 25 to 60 mm), two had a nodular pattern, four were diffuse, six showed patchy enhancement and one showed septal spread. Of the 14 patients with circumscribed malignancy, 13 had BCS and one had mastectomy. All four patients with diffuse malignancy had mastectomies. Five patients with types 2, 4 and 5 (nine patients in total) MRI patterns had mastectomies.

## Conclusion

MRI was helpful in the surgical decision where it showed either complete or partial therapeutic response in circumscribed malignancies, thereby avoiding unnecessary mastectomies. In diffuse malignancies, the decision was invariably mastectomy irrespective of the response to neoadjuvant chemotherapy.

